# Beyond the 150 min target: rethinking exercise promotion for active health in community-dwelling older adults through inclusive social prescribing

**DOI:** 10.3389/fpubh.2026.1877023

**Published:** 2026-06-22

**Authors:** Ning Kang, Chengmeng Zhang, Donghui Mei, Qiongyu Huang, Dongmin Wang

**Affiliations:** 1 Institute of Population Research, Peking University, Beijing, China; 2 Department of Physical Education, Peking University, Beijing, China

**Keywords:** active health, behavior change techniques, exercise promotion, healthy aging, social prescribing

## Abstract

Physical activity guidelines commonly recommend that older adults engage in at least 150–300 min of moderate intensity aerobic activity each week, combined with muscle strengthening and balance training. Although this target is clear, measurable, and useful for public health monitoring, it may be insufficient when directly applied to community-dwelling older adults who are frail, multimorbid, socially isolated, digitally excluded, or constrained by unsafe environments and limited care resources. For these populations, the central challenge is not only whether they can meet a weekly exercise volume, but whether they can preserve the functional abilities needed for independent living, social participation, and active aging. This Perspective argues for moving exercise promotion for community-dwelling older adults beyond a narrow adherence-based view of the 150-min target and toward a function-oriented paradigm of active health. We propose an integrated framework that places functional preservation at the center, uses micro dose exercise as an accessible entry point, applies Behavior Change Techniques to translate recommendations into sustainable daily habits, embeds exercise within community life through social prescribing, and supports long-term participation through human and digital collaboration. This approach does not reject existing physical activity guidelines. Rather, it reframes them as flexible references that can be adapted to older adults’ heterogeneous bodily conditions, social contexts, and everyday routines. By shifting from exercise adherence to long-term functional integration, community-based exercise promotion can become more inclusive, equitable, and feasible for older adults who are most likely to be left behind by standard dose-based models.

## 1 The tension between accelerated population aging and the limited reach of exercise guidelines

Global population aging has brought the question of how to help older adults become physically active to the center of the public health agenda. Almost all mainstream guidelines repeat a clear and powerful target: older adults should engage in at least 150–300 min of moderate intensity aerobic activity, or 75–150 min of vigorous intensity aerobic activity each week, together with muscle strengthening and balance training (1). This target is easy to communicate, monitor, and translate into policy. It has helped establish physical activity as a public health priority rather than a matter of personal preference.

However, the same target becomes less adequate when it is used as the dominant practical standard in community settings where older adults vary greatly in functional capacity, care resources, neighborhood safety, digital access, and social support (2, 3). In these situations, the 150-min target may function less as a supportive guide than as a threshold that many older adults cannot realistically cross (4). It tells them how much activity is recommended, but it says much less about where to start, how to continue, who should support them, and what kinds of exercise benefits matter most when functional decline is already present.

This gap is not only theoretical. Data from the 2024 National Health Interview Survey in the United States show that adherence to aerobic physical activity guidelines declines with age, with only 38.4 percent of adults aged 65 years and older meeting the relevant standard. Adherence also varies by income, education, disability status, and self-rated health (5, 6). The 150-min target is therefore not a neutral technical indicator in practice. It intersects with social position, bodily capacity, neighborhood conditions, digital access, and care networks (7). For an older person who lives alone, has knee pain, fears falling, and cannot use a smartphone, the problem is not simply lack of motivation (8). The problem is that the surrounding system may not make movement feasible, safe, or meaningful.

This perspective article argues that exercise promotion for community-dwelling older adults should move beyond a narrow adherence-based view of the 150-min target and toward a function-oriented paradigm of active health. The issue is not whether existing guidelines are wrong; rather, they are insufficient when treated as the main organizing logic of community practice. We propose a framework that places functional preservation at the center, uses micro dose exercise as an accessible entry point, applies Behavior Change Techniques, or BCTs, as the mechanism for habit formation, embeds activity within community life through social prescribing (i.e., a healthcare mechanism that connects individuals to non-clinical, community-based services to improve health and well-being), and supports long term participation through human digital collaboration. The aim is to shift exercise promotion from asking whether older adults meet a prescribed dose to asking whether movement can be made possible, meaningful, and sustainable in everyday life.

## 2 Why standard exercise promotion often misses community-dwelling older adults

A first limitation of conventional exercise promotion is its reliance on volume-based targets. The 150-min target is useful for population surveillance and public communication. However, as a practical intervention goal, it can obscure heterogeneity in later life (9). This heterogeneity is clinical, behavioral, and environmental. It includes differences in functional reserve, pain, chronic disease burden, caregiving responsibilities, digital literacy, and neighborhood resources (10). A single adherence threshold may therefore classify the most disadvantaged older adults as least successful, even when they face the greatest structural barriers to movement (11). This can unintentionally shift attention away from what they need most: safe movement, lower limb strength, balance, confidence, companionship, and accessible environments.

A second limitation is that many exercise programs report what was delivered, but not how behavioral change occurred (12). Community projects often include Tai Chi, walking groups, resistance training, health lectures, or online check ins (13, 14). These activities may be valuable, but their effects may depend on mechanisms beyond the activity itself. Older adults may participate because a routine has been created, instructors provide feedback, peers offer companionship, family members provide reminders, or the program renews their sense of being capable and socially valued (15). Without specifying such active ingredients, programs remain difficult to replicate, compare, and scale. The BCT taxonomy developed by Michie et al. (16) provides a shared language for identifying the active components of behavior interventions (17). The Behavior Change Wheel and the COM B model further indicate that behavior depends on capability, opportunity, and motivation (18, 19). This perspective is essential in later life, because inactivity rarely reflects willpower alone. It often reflects the combined limits of bodily capacity, environmental opportunity, social support, and perceived meaning.

A third limitation is the over-individualization of responsibility. Traditional exercise prescriptions often start from the message that older adults should exercise, control weight, or prevent disease (20). In real community settings, however, older adults may be inactive because no one accompanies them outdoors, walking paths are unsafe, transportation is limited, toilets and seats are unavailable, digital platforms are difficult to use, or they are not invited into social activities. Evidence on social prescribing shows that participation in community programs is shaped by physical limitations, digital exclusion, transport barriers, service location, and whether activities match personal interests (21). Exercise promotion should therefore be understood not as the outcome of individual discipline alone, but as part of a behavioral ecology formed by families, neighborhoods, community organizations, primary care, transportation, policy, and digital infrastructure.

## 3 A function-oriented framework for community-based exercise behavior promotion

### 3.1 Overview of the framework

The function-oriented paradigm proposed here does not reject the 150-min guideline. Instead, it changes the role of the guideline from a universal endpoint to a flexible reference. In this framework, the starting point is not a fixed subgroup label, but a functional question: which everyday abilities need to be protected, and what forms of movement can be safely and meaningfully embedded in daily life? The central object of intervention is therefore not exercising volume itself, but the preservation of functional abilities that allow older adults to stand up, walk safely, go outdoors, and participate in social life.

### 3.2 Functional preservation as the central goal

**Figure 1:**
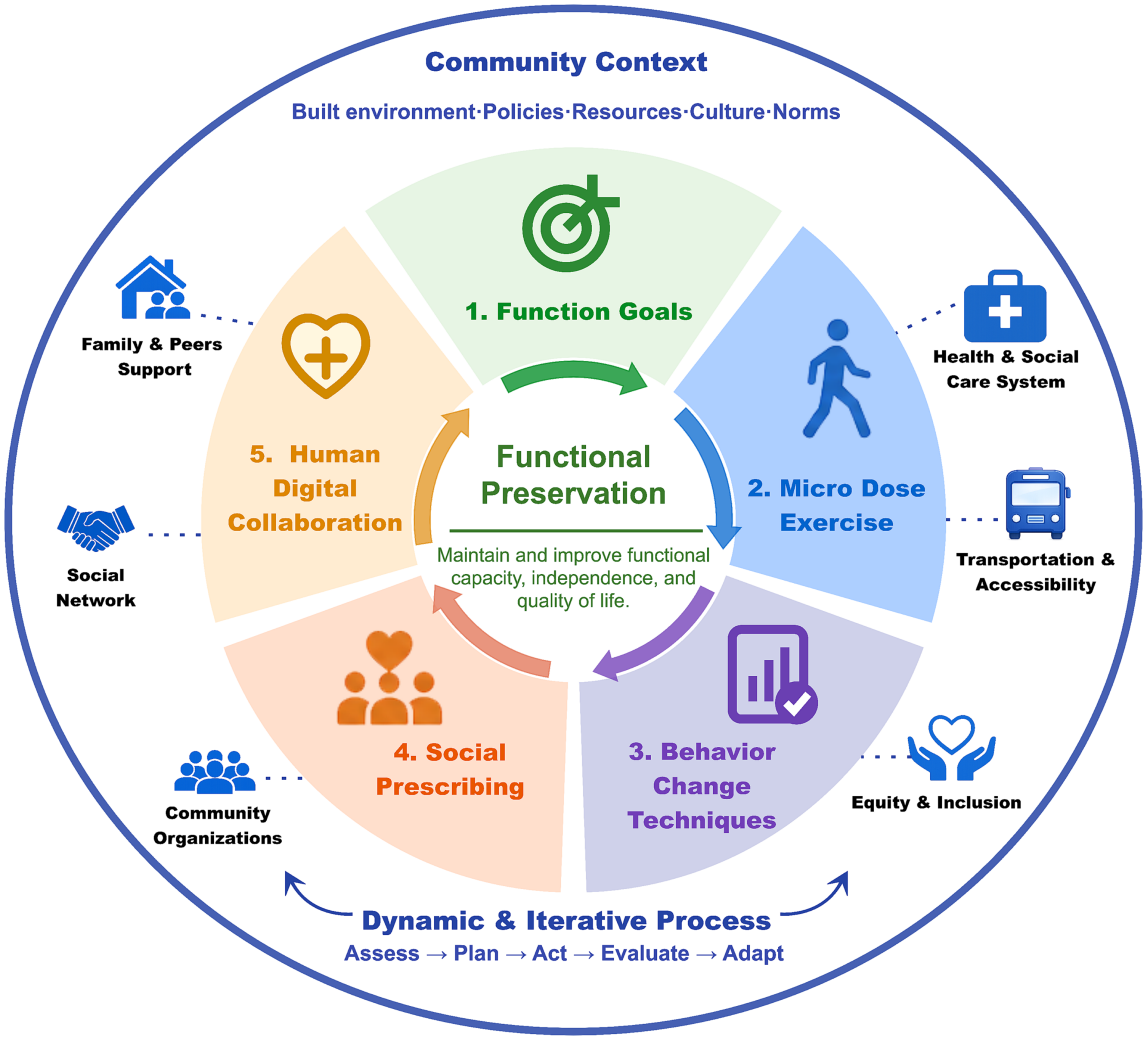
Conceptual framework for function-oriented exercise behavior promotion among community-dwelling older adults.

This framework consists of five interrelated components rather than a linear sequence. Functional preservation serves as the central goal. Micro-dose exercise provides the behavioral entry point. BCTs support the formation of sustainable routines. Social prescribing embeds movement in community resources and meaningful social roles. Human-digital collaboration provides continued support while reducing the risk of digital exclusion. As shown in Figure 1, these components are organized around functional preservation and work together to make movement more feasible, meaningful, and sustainable for community-dwelling older adults.

The intended outcome is sustained functional capacity within real life contexts. This can be understood as a form of functional symbiosis, in which older adults’ bodily abilities, daily routines, social relationships, and community environments mutually support one another over time (22, 23). In this sense, the framework does not aim simply to produce more older adults who meet exercise targets. It aims to build conditions under which movement becomes part of daily living, social connection, and active health.

Recent evidence supports this shift in emphasis. A prospective cohort study of 5,472 women aged 63–99 years assessed muscle strength using grip strength and the time needed to complete five sit to stand repetitions without using the hands, with an average follow up of 8.4 years. Better grip strength and sit to stand performance were associated with lower all-cause mortality risk, even after adjustment for moderate to vigorous physical activity, sedentary time, walking speed, and inflammatory indicators (24). This finding does not diminish the value of aerobic exercise. Rather, it suggests that for many older adults, the ability to rise safely from a chair may be closer to real survival capacity than completing a standard exercise session. Functional strength, balance, neuromuscular power, and movement confidence should therefore be placed at the center of community exercise promotion.

### 3.3 Micro-dose exercise as an accessible entry point

If functional preservation defines the goal, micro dose exercise offers a feasible entry point. Micro dose exercise refers to short, frequent, low threshold bouts of activity embedded in daily life (25). It should not be understood as a replacement for structured exercise, but as a practical pathway for those who cannot easily initiate conventional programs. A systematic review and meta-analysis of exercise snacks included 11 randomized controlled trials with 414 inactive adults and older adults (26). Exercise snacks were defined as structured activity bouts lasting no more than 5 min, performed throughout the day at least 3 days per week for at least 2 weeks. The review found improvements in cardiorespiratory fitness and some muscular fitness indicators.

The public health significance of this evidence lies in its potential for practical implementation. For older adults who have frailty, pain, fatigue, low confidence, limited time, unsafe environments, or limited social support, completing 30 min of continuous exercise may be difficult. In these situations, 2–5 min of chair-assisted sit-to-stand practice, marching in place after meals, heel raises during television breaks, or brief balance exercises before community activities may provide a more feasible starting point. These small actions change the basic unit of exercise promotion: instead of requiring a complete exercise session that depends on motivation, time, space, and planning, movement can be linked to familiar daily routines (27).

### 3.4 Behavior change techniques as behavioral scaffolding

However, small actions do not automatically become sustained habits. This is where BCTs provide behavioral scaffolding. Action planning helps older adults specify when, where, and what to do (16). Prompts and cues link movement to daily routines (28). Graded tasks allow frail individuals to begin from the smallest feasible dose (29). Demonstration and instruction reduce uncertainty and fear of injury (29). Self-monitoring and feedback make progress visible. Social support provides companionship, reminders, and safety. Problem solving helps older adults adapt to pain, bad weather, fatigue, caregiving duties, and space limitations (30, 31).

From the COM B perspective, these techniques are most useful when they address capability, opportunity, and motivation together (19). Capability includes physical ability to perform movements safely and psychological ability to understand and adapt them. Opportunity includes physical opportunity afforded by the environment (e.g., accessible spaces, seating, handrails, lighting, and transport) and social opportunity afforded by the cultural milieu, encompassing peer encouragement, social norms, and collective activity cues. Motivation includes beliefs about the value of movement, confidence in one’s ability, and the development of automatic routines. A community program that reports only the exercise type is therefore incomplete. A more useful report would specify which BCTs were used, who delivered them, in what setting, and how they supported capability, opportunity, and motivation. Such reporting would move community exercise promotion from activity delivery toward a more replicable public health intervention model.

### 3.5 Social prescribing as community embedding

Older adults are not isolated exercise performers. They are social actors living within families, neighborhoods, primary care systems, community organizations, and digital environments. Social prescribing is therefore central to the proposed framework. Its core function is to connect individuals with nonmedical community resources and with exercise classes, walking groups, arts, gardening, volunteering, and mutual support networks (32). A systematic review of older adults’ experiences suggests that social prescribing is a complex model of care designed to address nonmedical needs that are not adequately met by traditional health care, including loneliness, social isolation, low meaning, and low physical activity (33).

In exercise promotion, social prescribing transforms movement from a health task into a social role. Tai Chi, dance, gardening, and walking groups are not only physical activities. They also provide rhythm, cultural meaning, companionship, safety, responsibility, and belonging. This view is consistent with recent work on climate gerontology and green longevity (34, 35). These studies emphasizes that older adults should be recognized not only as vulnerable individuals, but also as active contributors to community resilience, environmental adaptation, and collective action (36, 37). In this sense, socially prescribed movement may help older adults rebuild agency, ecological connection, and social participation, rather than merely increase physical activity volume. Older adults may continue participating not because they have memorized a risk reduction message, but because they feel seen, needed, and connected. Social prescribing helps explain why older adults choose to engage in these physical activities (38). BCTs help them keep participating over time, while functional goals keep the focus on the abilities these activities are meant to protect.

Social prescribing is most likely to be effective when referral is accompanied by attention to access and fit. It requires attention to access and fit. Qualitative research identifies barriers such as physical limitations, digital exclusion, inaccessible transportation, and services concentrated in urban centers (21). Facilitators include personalized activities, digital support education, accessible venues, transportation arrangements, familiar community environments, and continued feedback. For older adults with mobility limitations, transportation support may matter more than wearable devices. For those with low digital literacy, face to face guidance may matter more than online classes. For lonely older adults, a stable companion may matter more than a personalized algorithm (21).

### 3.6 Human digital collaboration and equity

Digital health tools can support exercise promotion through reminders, records, video demonstrations, simple feedback, remote guidance, and risk alerts (39–43). They can also help community workers monitor participation and identify those who may need follow up. However, digital health is not automatically equitable. Research on older adults shows inequalities in digital access, use of health information technology, and self-efficacy in information seeking, and suggests that the digital divide may be associated with self-rated health (2, 44). If a community exercise program moves registration, check ins, feedback, and classes entirely to a mobile application, it may unintentionally exclude older adults with low income, low education, cognitive decline, sensory impairment, or solitary living.

A more equitable approach may be human digital collaboration rather than digital replacement. Digital tools could address low cost and repeatable tasks, such as reminders, recording, basic feedback, and simple demonstrations. Human support remains important for emotional companionship, movement correction, safety judgment, motivation, and meaning making. In this model, digital tools extend the support network rather than replace it. Health professionals, exercise instructors, occupational therapists, nurses, social workers, volunteers, family members, and community organizations should form the relational network that makes activity safe and meaningful. In this model, digital tools extend this network rather than replace it. For frail, cognitively vulnerable, or digitally disadvantaged older adults, digital interventions must be designed with robust accessibility features and integrated support, rather than relying solely on unguided digital formats that risk exacerbating the digital literacy gap (45).

### 3.7 Operationalizing functional preservation: starting points, progression, support, and outcomes

As Figure 1 indicates, functional preservation is the central outcome of the proposed framework. To ensure that this outcome remains measurable rather than abstract, functional preservation can be operationalized through simple, observable, and repeatable parameters. In this Perspective, “functional” refers to the capacity to perform essential daily movements that support independence, safety, and social participation, including standing up from a chair, walking safely, maintaining balance, climbing steps, carrying light objects, and going outdoors. These abilities can be assessed using practical indicators such as the five-times sit-to-stand test, usual gait speed, balance time, self-reported confidence in going outdoors, number of days per week with movement breaks, and participation in community activities.

A function-oriented approach may therefore begin with a simple baseline assessment rather than with the immediate expectation of meeting 150 min per week. For example, an older adult may start with one to three chair-assisted sit-to-stand repetitions, 1–2 min of marching in place, or a brief supported balance exercise linked to an existing routine such as after meals or before watching television. Progression can then be defined by small measurable increases, such as adding repetitions, extending the duration of balance practice, increasing the number of daily movement breaks, or improving confidence in walking outdoors. In this way, minutes and repetitions are not abandoned; rather, they are reframed as individualized indicators of functional progress.

Support is most useful when matched to need. Family members and peers can provide reminders and companionship; community workers and volunteers can assist with access, transport, and participation; exercise instructors, physiotherapists, occupational therapists, nurses, and primary care providers can assess risk, adapt movements, and monitor functional change; and digital tools can record repetitions, provide reminders, and visualize progress when appropriate. The most relevant benefits are not only increased activity volume, but also improved lower-limb strength, balance, mobility confidence, reduced fear of falling, ability to go outdoors, and sustained participation in community life. This approach preserves measurability while allowing exercise promotion to remain flexible for older adults with different functional capacities and social conditions.

## 4 Implications for public health governance and limitations

### 4.1 Implications for public health governance

Moving beyond the 150-min target has implications for policy and service design. First, medical insurance, long term care insurance, and public health funding could consider supporting or piloting community exercise care delivered through hybrid in person and digital models. Such care includes functional assessment, risk screening, movement instruction, behavior support, social connection, digital coaching, and family or volunteer accompaniment. These services are not drugs, surgery, or hospitalization, but they may help prevent functional decline and support aging in place.

Second, the enabling costs of social prescribing, such as transportation, accompaniment, accessible facilities, resting places, safe routes, and emergency assistance, may need to be considered in future funding models. Paying only for medical prescriptions while ignoring the conditions that allow participation may limit equity. Local policy experiments could explore support for community transportation, accompanied travel, activity link workers, and barrier free environments as part of health promotion or long-term care strategies.

Third, BCT coding could be incorporated into the design and evaluation of community exercise programs where feasible. Project proposals could define target behaviors, target populations, COM B barriers, BCT combinations, delivery agents, settings, and evaluation indicators. Although this may add design complexity, it could help avoid poorly specified programs in which activities appear well attended while the pathways through which older adults derive health benefits remain unclear.

Fourth, future guidelines could further move toward integrated twenty-four-hour behavior recommendations for older adults. The Canadian twenty-four-hour movement guidelines already place physical activity, sedentary behavior, and sleep within one framework (46). Recent research also suggests that physical activity, sedentary behavior, and sleep are interrelated with cognitive performance in older adults, and that sleep may shape the cognitive benefits of physical activity (47). Community exercise promotion may therefore benefit from addressing not only exercise, but also sedentary interruption, light activity, sleep rhythm, daytime structure, and cognitive maintenance.

### 4.2 Limitations and future directions

While the proposed framework has implications for public health governance, it should not be understood as a replacement for existing physical activity guidelines, clinical recommendations, or established evidence-based exercise programs. Rather, it is intended as a complementary framework for translating guideline-based recommendations into more feasible and equitable forms of action in community settings. Several limitations should therefore be acknowledged. First, the proposed framework is conceptual and practice-oriented rather than empirically validated as a complete intervention model. Future studies are needed to examine its feasibility, effectiveness, and implementation across different community settings. Second, although emerging evidence on exercise snacks and minimal-dose exercise is promising, the evidence base remains preliminary for frail older adults, older adults with multimorbidity, and those with cognitive or mobility limitations. Further intervention studies are needed to clarify safe starting doses, progression rules, supervision requirements, and long-term functional outcomes. Third, social prescribing is culturally and institutionally variable. Its implementation depends on local primary care systems, community organizations, transport resources, family structures, and norms of participation. Therefore, the framework should be understood as an adaptable model that requires contextual tailoring and further evaluation, rather than as a universal prescription.

## 5 Conclusion

Moving beyond the 150-min perspective does not mean abandoning physical activity guidelines. It means recognizing that guidelines must be translated into forms of action that are feasible, meaningful, and equitable for older adults with diverse functional capacities and social conditions. For community-dwelling older adults, the success of exercise promotion is better judged not only by whether a weekly volume target is achieved, but also by whether movement helps preserve independence, reduce functional vulnerability, strengthen social participation, and sustain active health in everyday life.

A function-oriented approach that integrates micro dose exercise, BCTs, social prescribing, and human digital collaboration offers a practical pathway toward this goal. It helps reframe older adults not as passive recipients of prescriptions or as individuals who fail to comply with guidelines, but as people whose capacity to move depends on the fit between bodily ability, daily routine, social relationships, and community environment. The future of exercise promotion in aging societies may be better measured not only by how many older adults meet standardized exercise volume targets, but also by measurable indicators of functional capacity and participation, such as the ability to rise from a chair, walk safely, maintain balance, go outdoors with confidence, and remain connected to community life.

## Data Availability

The original contributions presented in the study are included in the article/supplementary material, further inquiries can be directed to the corresponding author.
